# Antipsychotic medication use and fracture: a case–control study

**DOI:** 10.1038/s41598-023-40762-w

**Published:** 2023-08-22

**Authors:** Behnaz Azimi Manavi, Amanda L. Stuart, Julie A. Pasco, Jason M. Hodge, D. Kavindi Weerasinghe, Rasika M. Samarasinghe, Lana J. Williams

**Affiliations:** 1 IMPACT – the Institute for Mental and Physical Health and Clinical Translation, School of Medicine, Deakin University, Geelong, 3220 Australia; 2 Barwon Health, Geelong, 3220 Australia; 3 Department of Medicine-Western Health, The University of Melbourne, St Albans, 3021 Australia; 4 Department of Epidemiology and Preventive Medicine, Monash University, Melbourne, 3004 Australia

**Keywords:** Diseases, Medical research

## Abstract

It has been reported that antipsychotic use is associated with lower bone mineral density and bone quality. We aimed to determine whether antipsychotic use is associated with fracture risk in a population-based sample of adults living in the Barwon Statistical Division, south-eastern Australia. In this case–control study, 1458 participants (51.8% women) with radiologically confirmed fracture between June 1st 2012 and May 31st 2013 (cases) were compared with 1795 participants (46.5% women) without fracture (controls) for the same time period. Medication use, medical history and lifestyle factors were documented by self-report. Multivariable binary logistic regression was used to explore associations between antipsychotic use and fracture following adjustment for possible confounders. In women, antipsychotic use was identified for 20 of 755 (2.6%) cases and 10 of 834 (1.2%) controls (*p* = 0.034) and in men, antipsychotic use was identified for 13 of 703 (1.8%) cases and 5 of 961 (0.5%) controls (*p* = 0.010). Following adjustments, antipsychotic use was associated with a 3.0-fold increased risk of fracture in men and a 2.3-fold increased risk of fracture in women. Patterns persisted after exclusion of participants with non-fragility fractures and self-reported schizophrenia. While future research exploring underlying mechanisms is needed, regular monitoring of bone health in antipsychotic users is suggested.

## Introduction

Antipsychotic medications are used primarily in the treatment of schizophrenia and bipolar disorder^1^. The prescribing of antipsychotics has increased globally^2^. The number of Australians prescribed one or more antipsychotics increased from approximately 261,000 in 2005 to 422,000 in 2021^3^. Off-label use of antipsychotics has also grown, with 40–75% of all prescribed antipsychotics among adults for indications including mood and anxiety disorders, insomnia and agitation. Among children, off-label use accounted for 36–93% of all prescribed antipsychotics for indications including attention-deficit-hyperactivity-disorder (ADHD), anxiety and mood disorders^4^. Concerns have been raised regarding the long-term safety profile of antipsychotics^5^, with side effects including extrapyramidal symptoms, orthostatic hypertension, hyperprolactinemia, sedation, sexual dysfunction and possibly osteoporosis^5,6^.

Osteoporosis is “a skeletal disorder characterised by compromised bone strength that increases the risk of fracture”^7^. Osteoporosis is becoming a significant public health concern, being the most common bone disease in humans^8^. In Australia, 12.4% of general practice patients aged over 50 years, have osteoporosis^9^. Osteoporosis is a silent disease and is often not diagnosed until a fracture occurs^10^. It has been reported that individuals with a prior fracture are at higher risk of subsequent fractures^10,11^, leading to reduced quality of life and wellbeing, short-term morbidity, higher disability rates and related hospital admissions^12^. It was estimated that in 2013, 395 fractures occur each day in Australia, with this rate having been estimated to increase to 501 fractures per day by 2022^10^. The consequences of a fracture are not limited to a person’s health and wellbeing, there is also a heavy financial burden on the health system. In Australia, the total direct cost of treatment associated with osteoporosis was estimated to be $3.44 billion in 2017, which is three times higher than the reported cost in 2007^13^. Thus, raising awareness to promote bone health and reduce fracture-related consequences and costs is needed.

A growing body of work has investigated fracture risk in patients taking antipsychotics, with these being limited to clinical groups such as hospitalised patients with dementia^14^ or patients with schizophrenia^15^. Following adjustment for a diagnosis of schizophrenia, some studies^16–19^ but not all^20,21^, have observed an independent association between antipsychotic medication and hip fracture. Furthermore, few population-based studies have evaluated the risk of any fracture in long-term users of antipsychotics and those that have, are limited to elderly populations^22–28^. To bridge this gap, we conducted this study to investigate the association between antipsychotic use and fracture risk in a large population-based sample of men and women aged 20 years and older.

## Materials and methods

### Study region

Fracture cases and non-fracture controls were all recruited from the Barwon Statistical Division (BSD), situated in south-eastern Australia. The BSD is geographically a clearly defined region comprising a well-characterised population that is representative of the broader Australian population in terms of age distribution, education, marital status, employment and income profiles^29^.

### Participants

Fracture cases: All adults (aged 20 years or over) living in the BSD who sustained a fracture at any skeletal site between June 1st 2012 and May 31st 2013 were invited to participate in the PRedictors and Outcomes of incident FRACtures (PROFRAC) study, a case–control study designed to examine predictors and consequences of incident fracture in men and women. Fractures were identified by a daily computerised keyword search of all radiological reports at the University Hospital Geelong (Barwon Health). Exclusion criteria included fractures with a pathological origin and/or inability to provide written informed consent. In total, 1458 participants (51.8% women) with a radiologically confirmed fracture agreed to participate, with 67.7% response. Further details of sample selection, recruitment, methodology and inclusion/ exclusion criteria have been published elsewhere^30^.

Non-fracture controls: non-fracture controls identified as free of incident fracture between June 1st 2012 and May 31st 2013 were drawn from the Geelong Osteoporosis Study (GOS), an ongoing, population-based cohort study^29^. In total, 1494 women aged 20–94 years (77.1% participation) were recruited during 1994–1997, and 1540 men aged 20–93 years (67.1% participation) were recruited during 2001–2006. Participants have returned for follow up every 2–5 years. Detailed sample recruitment details have been published elsewhere^29^. For these analyses, data were drawn from the 5-year follow-up for men (2005–2006) and the 15-year follow-up for women (2011–2014). A total of 1,795 participants (46.5% women) were included as controls.

All participants provided informed, written consent. Ethics approval was obtained from the Barwon Health Human Research Ethics Committee.

### Data collection

The following data were collected at the time of fracture for the cases and time of assessment for the non-fracture controls.

### Fracture

Using a previously validated method of fracture ascertainment^31,32^, individual with incident fractures were identified by a computerised keyword search of all radiological reports at the University Hospital Geelong (Barwon Health) between June 1st 2012 to May 31st 2013 for the cases and controls. Based on this method, “possible” or “suggested” fractures were captured but not considered unless the fracture was later confirmed through subsequent radiological reports^31^.

### Medications

Exposure to medications and duration of use were documented by questionnaire. Medications pertinent for these analyses included antipsychotics (typical and atypical), other psychotropics (antidepressants, anticonvulsants, antianxieties, antiemetics and/or antinauseants, sedatives and/or hypnotics, movement disorder agents) and bone related medications known to affect bone positively (antiresorptives/bisphosphonates, hormone therapy, and calcium and vitamin D supplements) or negatively (oral glucocorticoids and thyroid hormones) that were used at the time of assessment for both the cases and controls. For cases, medications also needed to be taken at least one month prior to fracture to be considered.

### Lifestyle and health factors

Data on smoking, alcohol consumption, physical activity, previous fracture, falls and history of schizophrenia were documented by self-report. Smoking at the time of assessment was considered current. Frequency of alcohol consumption was determined using a five-point scale (never, less than once per week, once or twice per week, several times per week, and every day) and categorised as frequent (several times per week or every day) or infrequent use (never, less than once per week, once or twice per week). Metabolic Equivalent of Task values were used to group participants as active (very active or active) or inactive (sedentary, limited activity, or chair or bedridden)^29,33^. Previous fracture was considered for those who had sustained a fracture prior to June 1st 2012. Falling was considered if at least one fall to the ground had occurred during the 12 months prior to assessment. History of schizophrenia was self-reported.

Height and weight were self-reported for cases. For controls, height was measured using a Harpenden stadiometer and weight using electronic scales. Body Mass Index (BMI) was calculated as weight/height^2^ (kg/m^2^). Based on World Health Organization criteria, a BMI of 25 kg/m^2^ and over was considered overweight^34^.

## Statistical analysis

Mean and standard deviation (SD) were reported for continuous parametric variables, median and interquartile ranges (IQR) for continuous non-parametric variables and counts and percentages were provided for categorical variables. Inter-group differences between cases and controls were analysed using t-tests for parametric data, Mann–Whitney for non-parametric data and the Chi-Square test for categorical data.

Binary logistic regression [odds ratio (OR), with 95% confidence intervals (CI)] was used to investigate the association between antipsychotic use and fracture. Unadjusted (model I), age and BMI adjusted (model II) and fully adjusted models for additional known risk factors including smoking, activity, alcohol consumption, previous fracture, falls and medications known to affect bone (model III) are presented. Separate models were generated for men and women. Potential interactions between exposure variables and antipsychotic use were tested in the fully adjusted models. Two sensitivity analyses were performed: one following removal of fractures of the finger/thumb, toe, face, skull, patella and clavicle (non-fragility fractures) and the other after removing participants with self-reported schizophrenia. SPSS statistical package 28 for windows (SPSS Inc., Chicago, IL, USA) was used for statistical analyses.

### Ethics approval and consent to participate

Ethics approval was obtained from the Human Research Ethics Committee at Barwon Health (ID 92/01 and 00/56). All participants provided informed, written consent.

### Human and animal rights

All procedures performed in studies involving human participants were in accordance with the ethical standards of the institutional and/or national research committee and with the 1964 Helsinki declaration and its later amendments or comparable ethical standards.

## Results

### Men

There were 703 cases with incident fracture and 961 controls. Fracture sites by age groups are presented in Table 1. Cases were younger, had lower BMI and were more likely to smoke, drink alcohol less frequently, use antipsychotics, other psychotropics, and gonadal hormones; otherwise, the groups were similar in regard to activity level, previous fracture, falls, self-reported schizophrenia, and use of adrenal steroid hormones, thyroid hormones, anti-fracture agents, and calcium and vitamin D (Table 2).

**Table 1:** Fracture site by age group. Results are displayed as n (%).

Fracture sites	Men					Women				
	Age categories				Total	Age categories				Total
	20–39	40–59	60–79	80–100		20–39	40–59	60–79	80–100	
	n = 270	n = 221	n = 147	n = 65	n = 703	n = 119	n = 192	n = 291	n = 153	n = 755
Face	16 (3.9%)	11 (2.0%)	5 (1.0%)	1 (0.5%)	33 (2.0%)	6 (2.1%)	6 (1.2%)	2 (0.4%)	2 (0.9%)	16 (1.1%)
Skull	2 (0.5%)	0 (0.0%)	3 (0.6%)	0 (0.0%)	5 (0.3%)	0 (0.0%)	0 (0.0%)	4 (0.7%)	0 (0.0%)	4 (0.3%)
Vertebra	6 (1.4%)	11 (2.0%)	27 (5.5%)	14 (6.0%)	58 (3.5%)	2 (0.6%)	5 (1.0%)	34 (5.8%)	26 (11.3%)	67 (4.2%)
Rib, sternum	11 (3.6%)	24 (4.3%)	21 (4.3%)	8 (3.9%)	64 (3.8%)	3 (1.0%)	5 (1.0%)	12 (2.1%)	10 (4.3%)	30 (2.0%)
Pelvis	3 (0.7%)	4 (0.7%)	1 (0.2%)	7 (3.4%)	15 (0.9%)	3 (1.0%)	0 (0.0%)	14 (2.4%)	10 (4.4%)	27 (1.7%)
Collar bone (clavicle)	21 (5.0%)	15 (2.7%)	6 (1.2%)	1 (0.5%)	43 (2.7%)	5 (1.7%)	4 (0.8%)	3 (0.5%)	1 (0.4%)	13 (0.8%)
Shoulder blade (scapula)	6 (1.4%)	1 (0.2%)	1 (0.2%)	1 (0.5%)	9 (0.5%)	0 (0.0%)	1 (0.2%)	1 (0.2%)	0 (0.0%)	2 (1.0%)
Upper arm (humerus)	10 (2.4%)	5 (0.9%)	8 (1.6%)	6 (3.0%)	29 (1.8%)	3 (1.0%)	11 (2.3%)	29 (5.0%)	18 (7.9%)	61 (3.9%)
Forearm (radius and ulna)	29 (7.0%)	8 (1.5%)	5 (1.0%)	2 (1.0%)	44 (2.7%)	10 (3.4%)	17 (3.5%)	17 (2.9%)	7 (3.0%)	51 (3.2%)
Wrist (Colles’/Smith’s)	18 (4.3%)	20 (3.6%)	19 (3.8%)	5 (2.5%)	62 (3.7%)	19 (6.5%)	38 (7.9%)	67 (11.4%)	33 (14.3%)	157 (9.9%)
Wrist (Carpal bones)	20 (4.8%)	5 (0.9%)	6 (1.2%)	1 (0.5%)	32 (2.0%)	2 (0.7%)	4 (0.8%)	3 (0.5%)	0 (0.0%)	9 (0.5%)
Hand (metacarpal bones)	38 (9.1%)	10 (1.9%)	2 (0.4%)	1 (0.5%)	51 (3.1%)	3 (1.0%)	2 (0.4%)	2 (0.4%)	1 (0.4%)	8 (0.5%)
Finger/thumb	37 (8.9%)	42 (7.6%)	11 (2.2%)	2 (1.0%)	92 (5.6%)	11 (3.8%)	14 (2.9%)	9 (1.5%)	1 (0.4%)	35 (2.2%)
Hip (cervical)	1 (0.2%)	5 (0.9%)	7 (1.4%)	13 (6.4%)	26 (1.6%)	1 (0.3%)	2 (0.4%)	27 (4.6%)	37 (16.1%)	67 (4.2%)
Upper leg (other parts of femur)	4 (1.0%)	4 (0.7%)	4 (0.8%)	3 (1.5%)	15 (0.9%)	0 (0.0%)	1 (0.2%)	3 (0.5%)	6 (2.6%)	10 (0.6%)
Knee cap (patella)	3 (0.7%)	1 (0.2%)	7 (1.4%)	3 (1.5%)	14 (0.9%)	2 (0.7%)	3 (0.6%)	5 (0.9%)	4 (1.7%)	14 (0.9%)
Lower leg (tibia and fibula)	14 (3.3%)	15 (2.7%)	5 (1.0%)	1 (0.5%)	35 (2.1%)	3 (1.0%)	12 (2.5%)	11 (1.8%)	3 (1.3%)	29 (1.8%)
Ankle	21 (5.1%)	33 (6.0%)	12 (2.4%)	2 (1.0%)	68 (4.2%)	14 (4.8%)	38 (7.9%)	36 (6.1%)	9 (3.9%)	97 (6.1%)
Foot (tarsal and metatarsal bones)	21 (5.1%)	23 (4.1%)	7 (1.4%)	1 (0.5%)	52 (3.1%)	27 (9.3%)	29 (6.0%)	23 (3.9%)	3 (1.3%)	82 (5.2%)
Toe	7 (1.6%)	13 (2.3%)	5 (1.0%)	1 (0.5%)	26 (1.6%)	12 (4.1%)	6 (1.3%)	13 (2.2%)	1 (0.4%)	32 (2.0%)

**Table 2:** Characteristics of fracture cases and controls for men and women. Results are displayed as median (interquartile range), mean ± SD or n (%).

	Men			Women		
	Cases	Controls	*p*	Cases	Controls	*p*
	n = 703	n = 961		n = 755	n = 834	
Age (year)	48.5 (31.8–62.7)	59.5 (45.9–73.4)	< 0.001	61.7 (49.2–76.7)	56.3 (42.5–69.7)	< 0.001
BMI (> 25 kg/m^2^)	418 (59.5%)	670 (69.7%)	< 0.001	374 (49.5%)	526 (63.1%)	< 0.001
Smoking (current)	152 (21.8%)	108 (11.3%)	< 0.001	95 (12.8%)	91 (11.1%)	0.295
Activity (current)	522 (75.3%)	677 (71.0%)	0.050	449 (60.1%)	578 (70.8%)	< 0.001
Frequent alcohol consumption (current)	222 (31.6%)	394 (41.0%)	< 0.001	180 (23.8%)	186 (22.3%)	0.467
Previous fracture (prior to June 1st 2012)	246 (36.5%)	318 (34.2%)	0.340	276 (37.7%)	155 (18.7%)	< 0.001
Faller (past 12 months)	111 (16.0%)	172 (18.1%)	0.274	177 (23.7%)	224 (27.1%)	0.123
Schizophrenia (lifetime)	7 (1.0%)	3 (0.3%)	0.107	2 (0.3%)	1 (0.1%)	0.607
Medication use (current)						
Antipsychotics	13 (1.8%)	5 (0.5%)	0.010	20 (2.6%)	10 (1.2%)	0.034
Other psychotropics	132 (18.8%)	100 (10.4%)	< 0.001	246 (32.6%)	200 (24.0%)	< 0.001
Adrenal steroid hormones	15 (2.1%)	10 (1.0%)	0.070	31 (4.1%)	23 (2.8%)	0.139
Gonadal hormones	9 (1.3%)	3 (0.3%)	0.036	37 (4.9%)	32 (3.8%)	0.299
Thyroid hormones	7 (1.0%)	10 (1.0%)	0.928	58 (7.7%)	61 (7.3%)	0.781
Anti-fracture agents	18 (2.6%)	17 (1.8%)	0.266	58 (7.7%)	28 (3.4%)	< 0.001
Calcium/vitamin D supplements	50 (7.1%)	62 (6.5%)	0.595	219 (29.0%)	190 (22.8%)	0.005

Thirteen (1.8%) cases used antipsychotics [Quetiapine (n = 8), Olanzapine (n = 1), Risperidone (n = 1), Aripiprazole (n = 1), Chlorpromazine (n = 1), Clozapine (n = 1)] versus five (0.5%) controls [Olanzapine (n = 3), Quetiapine (n = 1), Risperidone (n = 1)]. Median duration of antipsychotic use between the cases and controls did not differ [45.0 (IQR 29.0–77.0) vs. 74.0 (IQR 4.0–92.0) months, *p* = 0.859].

Table 3 presents the multivariable logistic regression models for the association between antipsychotic use and fracture. Antipsychotic use was associated with an increased risk of fracture before (model I) and after adjustment for age and BMI (model II), with this relationship persisting in the fully adjusted model (model III). Age, BMI, smoking, alcohol consumption and medications known to affect bone were independently associated with fracture.

**Table 3:** Unadjusted, age- and BMI-adjusted and fully adjusted logistic regression models presenting the association between antipsychotic use and fracture.

	Men						Women					
	Model I		Model II		Model III		Model I		Model II		Model III	
	OR (95% CI)	*p*	OR (95% CI)	*p*	OR (95% CI)	*p*	OR (95% CI)	*p*	OR (95% CI)	*p*	OR (95% CI)	*p*
Antipsychotic use	3.60 (1.28–10.15)	0.015	3.60 (1.24–10.45)	0.020	3.00 (1.00–8.90)	0.056	2.24 (1.04–4.82)	0.040	2.40 (1.09–5.26)	0.030	2.29 (1.02–5.13)	0.044
Age	–	–	0.96 (0.96–0.97)	< 0.001	0.96 (0.96–0.97)	< 0.001	–	–	1.02 (1.01–1.02)	< 0.001	1.01 (1.00–1.02)	< 0.001
BMI (> 25 kg/m^2^)	–	–	0.70 (0.57–0.87)	0.001	0.74 (0.59–0.93)	0.010	–	–	0.54 (0.44–0.67)	< 0.001	0.56 (0.45–0.70)	< 0.001
Smoking	–	–	–	–	1.55 (1.16–2.10)	0.003	–	–	–	–	1.15 (0.83–1.61)	0.401
Activity	–	–	–	–	0.88 (0.68–1.14)	0.344	–	–	–	–	0.72 (0.56–0.93)	0.011
Alcohol consumption	–	–	–	–	0.73 (0.59–0.91)	0.005	–	–	–	–	0.96 (0.74–1.23)	0.740
Previous fracture	–	–	–	–	1.20 (0.96–1.50)	0.110	–	–	–	–	2.40 (1.90–3.06)	< 0.001
Faller	–	–	–	–	0.95 (0.72–1.27)	0.72	–	–	–	–	0.69 (0.54–0.90)	0.004
Medications known to affect bone positively	–	–	–	–	1.66 (1.10–2.50)	0.015	–	–	–	–	1.10 (0.86–1.40)	0.443
Medications known to affect bone negatively	–	–	–	–	2.90 (1.50–5.65)	0.002	–	–	–	–	1.04 (0.73–1.47)	0.830

### Women

There were 755 cases with incident fracture and 834 controls. Fracture sites by age groups are presented in Table 1. Cases were older, had lower BMI, were less active and were more likely to have had a previous fracture, and use antipsychotics, other psychotropics, anti-fracture agents and calcium and vitamin D; the groups were similar in regard to smoking, alcohol consumption, falls history, self-reported schizophrenia and use of adrenal steroid hormones, gonadal hormones, and thyroid hormones (Table 2).

Twenty (2.6%) cases used antipsychotics [Flupentixol (n = 1), Quetiapine (n = 9), Olanzapine (n = 8), Ziprasidone (n = 1), Aripiprazole (n = 1)] versus ten (1.2%) controls [Quetiapine (n = 7), Aripiprazole (n = 1), Olanzapine (n = 1), Paliperidone (n = 1)]. Median duration of antipsychotic use between the cases and controls did not differ [28.0 (IQR 19.0–32.0) vs. 42.0 (IQR 13.0–82.0) months, *p* = 0.308].

Table 3 presents the multivariable logistic regression models for the association between antipsychotic use and fracture. Antipsychotic use was associated with an increased risk of fracture before (model I) and after adjustment for age and BMI (model II), with this relationship persisting in the fully adjusted model (model III). Age, BMI, activity, previous fracture and falls were independently associated with fracture.

### Sensitivity analyses

Patterns persisted after excluding 325 participants with fractures of the finger/thumb, toe, skull, face, patella and clavicle (age- and sex-adjusted OR 2.14, 95% CI 1.20–3.80, *p* = 0.010).

In a second sensitivity analysis, after excluding 13 participants with self-reported schizophrenia, patterns also persisted (age- and sex-adjusted OR 2.53, 95% CI 1.27–5.05, *p* = 0.008).

## Discussion

In this case–control study spanning the adult age spectrum, our results demonstrate that exposure to antipsychotic medication is associated with increased fracture risk women, with this pattern also being observed for men. After taking potential confounders into consideration, antipsychotic use was associated with a 2.3-fold greater risk of fracture for women and a 3.0-fold increased risk of fracture for men.

Similar to our findings, Hugenholtz et al.^17^ utilising data from the UK General Practice Research Database (n = 44,500, 75.8% women) found antipsychotic use was associated with a 1.3-fold greater risk of hip fracture compared to controls. Similarly, Bolton et al.^35^ using data from the Manitoba Bone Density Program database (n = 68,730, 90.6% women) found antipsychotic use to be independently associated with a 1.4-fold increased risk of major osteoporotic fractures and a 2.1 increased risk of hip fracture compared to non use. Further support comes from a meta-analyses whereby antipsychotic use was consistently shown to increase risk of any fracture by 1.2–1.6 fold, albeit in older adults^6,36–38^.

Others have explored the association between antipsychotics use and fracture in patients with schizophrenia. Howard et al.^16^ conducted a case–control study investigating the association between schizophrenia and hip fracture, using data from the General Practice Research Database. Comparing 16,341 (79% women) cases with hip fracture and 29,889 (79% women) controls without fracture, antipsychotic use was independently associated with hip fracture, while schizophrenia was not^16^. Sorensen et al.^18^ using a Danish hospital register comprising 15,431 (41% women) patients with schizophrenia and 3,807,597 (57% women) controls found that after adjusting for a diagnosis of schizophrenia, antipsychotic use was associated with a 1.2-fold increased rate of hip fracture. Similarly, Tsai et al.^39^ using the National Health Insurance Research Database compared 30,335 (50.1% women) patients with schizophrenia with 121,340 (50.1% women) controls and found a diagnosis of schizophrenia was not associated with major osteoporosis fracture, while antipsychotic use was. Conversely, Bolton et al.^20^ using an administrative database of 15,792 (70.3% women) patients with fractures and 47,289 (70.2% women) matched controls and found that a diagnosis of schizophrenia explained the association between antipsychotic use and fracture. However, in the current study we had a small number of participants with self-reported schizophrenia; after removing those with schizophrenia, the association between antipsychotic use and fracture remained.

Antipsychotic use may increase the risk for fracture through several mechanisms. Antipsychotics that block dopamine-D2 receptors, which consequently inhibit the action of dopamine on prolactin secretion resulting in hyperprolactinemia. This can adversely affect bone cell metabolism and accelerate rate of bone loss, thereby increasing fracture risk^40,41^. Interestingly, not all antipsychotics raise prolactin levels, thus other mechanisms must be at play. It has been reported that 90% of fractures, particularly hip and femur fractures, are related to falls^32,42^. Antipsychotic use has previously been associated with a higher risk of falls^43–45^, possibly due to orthostatic hypotension or sedation^5^, a well-known risk factor for increasing fall-related fractures^46^. Furthermore, the use of antipsychotics has been associated with decreased bone mineralisation^47^, and bone quality^48^ leading to weaker bones^49^ and a higher probability that a fall will result in a fracture.

Our findings align with previous studies reporting a higher risk of fracture in men using antipsychotics^16,27,50,51^. The sex differences may relate to higher prolactin with associated hypogonadism having a profound effect on male compared to female bone^52,53^. However, data related to prolactin were not collected in this study. In this study, a higher proportion of women with fracture were taking calcium and vitamin D supplements and anti-fracture medication compared with their male counterparts; all protective factors against fracture. Although, these factors were tested in the models and did not explain the findings.

A major strength of this study is that the relationship between antipsychotic use and fracture risk was tested within a population-based sample of men and women, spanning the full adult age range. Previous research has mostly been conducted in elderly populations and in patients with a diagnosis of schizophrenia. In addition, the design of this study enabled us to account for several potential confounding factors. Nevertheless, this study has several limitations that need to be acknowledged. First, the small number of antipsychotic users prevented sub-group analyses examining specific antipsychotic agents and the role of duration and dose in association with fracture risk. Second, this study did not explore a mechanism of action such as the role of prolactin in this association. Third, potential unrecognised confounding may affect our results. Fourth, weight and height were self-reported for cases which may lead to inaccuracies. Fifth, while the median duration of antipsychotic use was not significantly different for cases and controls, we should acknowledge that the difference could be clinically relevant. Sixth, although controls were fracture free for the same time period as the fracture cases, all other data were drawn from the closest GOS follow up appointment; hence, time-related biases may be at play^54^. Seventh, other psychotropic use could not be tested in the models due to collinearity. Eighth, due to the limited number of people with schizophrenia and non-fragility fractures, data was pooled for each sensitivity analyses. Last, participants were largely Caucasian, so our findings may not be generalisable to other populations.

## Conclusion

Our results demonstrate that exposure to antipsychotic medication is associated with increased fracture risk women, with this pattern also being observed for men. Assessment of bone health among antipsychotic users should be considered, with early detection and management of bone loss likely to reduce fracture risk. Future research investigating the underlying mechanism in the association between antipsychotic use and fracture is warranted.

## Data Availability

The datasets generated during and/or analysed during the current study are available from the corresponding authors on reasonable request.
